# Artificial Intelligence Attitudes and Time Management Among Undergraduate Nursing Students: A Cross‐Sectional Study

**DOI:** 10.1155/jonm/7221445

**Published:** 2026-03-25

**Authors:** Ramazan Bozkurt, Musa Can Özdoğan

**Affiliations:** ^1^ Faculty of Health Sciences, Nursing Department, Fundamentals of Nursing, Sakarya University, Sakarya, 54050, Türkiye, sakarya.edu.tr; ^2^ Institute of Health Sciences, Master Science of Nursing Program, Sakarya University, Sakarya, 54050, Türkiye, sakarya.edu.tr

**Keywords:** artificial intelligence, nursing, skills, students, time management

## Abstract

**Background:**

Artificial intelligence (AI) has emerged as an innovative technology that is increasingly used in healthcare. In the nursing profession, AI offers several advantages, including time savings through accelerated care processes, reduced error rates, and enhanced patient safety.

**Aim:**

This study aimed to describe undergraduate nursing students’ attitudes toward AI and their time management skills and to examine the relationship between these variables.

**Methods:**

This cross‐sectional study was conducted with 360 undergraduate nursing students enrolled at a state university in northwestern Turkey. The data were collected between September and December 2024 using the Individual Identification Form, the General Attitude Scale Towards AI, and the Time Management Questionnaire. The study was conducted in accordance with the principles of the Declaration of Helsinki.

**Results:**

Among participants, 58.9% were aged 21 years and above, 83.3% were female, and 32.5% were first‐year students. The mean score on the General Attitudes Toward AI Scale was 68.92 ± 9.21, while the mean score on the Time Management Questionnaire was 85.02 ± 12.61. A statistically significant positive correlation was found between attitudes toward AI and time management skills (*r* = 0.119, *p* < 0.05).

**Conclusions:**

Nursing students demonstrated high levels of positive attitudes toward AI and strong time management skills. As students’ attitudes toward AI increased, their time management skills also improved. Further controlled and longitudinal studies are needed to confirm the mechanisms and long‐term effects of AI‐based time efficiency. Integrating AI literacy and ethical use modules into undergraduate nursing curricula is recommended to promote the responsible and effective use of AI tools in the context of patient safety and academic integrity.

## 1. Background

Artificial intelligence (AI) is an information system that treats knowledge as an object—acquiring, analyzing, and representing it to simulate human intellectual activity [1]. It also refers to modeling the intelligent behavior of computers with minimal human intervention [2]. According to nursing researchers Fritz and Dermody [3], AI is defined as “a computer algorithm that acts as a rational agent capable of assessing human movement over time and making decisions about that movement, much like a human would.”

The nursing profession, positioned at the core of healthcare delivery, is advancing alongside technological developments in AI. The use of AI in healthcare enables clinical predictions, such as monitoring disease progression, identifying risks, implementing preventive measures, and making forecasts through machine learning [4]. In addition to its potential benefits for healthcare professionals—including nurses and midwives—AI offers applications that address patient‐related risks and system limitations [5, 6]. Moreover, AI is increasingly utilized in nursing practice, education, research, and policy [7, 8].

AI contributes to various aspects of healthcare, including performing physically demanding care procedures, supporting mental health, predicting risks and diseases, empowering patients, preventing injuries, and improving cost‐efficiency through early diagnosis. It also assists in developing treatment plans, preparing medication lists, enhancing surgical performance, guiding clinical decision‐making, and reducing practitioners’ workloads [9–11]. Given its capacity to save time, optimize efficiency, automate processes, and streamline organizational management, it is essential that nurses and nursing students adopt and utilize this innovative technology [12–14]. However, a systematic review indicates that nearly half of studies (48.57%) report that nurses and midwives are not currently involved in the development, implementation, or evaluation of AI systems, even though they are expected to be future users of such technologies [5].

To ensure the effective and sustainable use of AI technologies in nursing practice, existing health policies and legal frameworks require comprehensive review and revision. The successful integration of AI into nursing practice necessitates the incorporation of supportive regulatory frameworks and standardized guidelines into health policy structures [15]. Furthermore, enabling nurses to use AI‐based tools effectively underscores the critical need to transform and align nursing education programs with these emerging technologies through systematic curricular restructuring [16].

To prepare for these transformations, nursing students should acquire the knowledge and skills needed to use AI tools effectively, evaluate their reliability, and integrate appropriate technologies safely into clinical settings [17, 18]. Encouraging students to engage with AI applications in nursing practice can enhance the efficiency of healthcare delivery and improve patient outcomes [19]. Positive attitudes toward AI among nursing students have been shown to influence their intention to adopt these technologies [20]. Recent studies report generally positive attitudes among nursing students toward AI [16, 21, 22]. Evidence from both national and international literature indicates that students generally demonstrate a high level of awareness of AI and tend to develop more positive attitudes when they perceive the potential contributions of these technologies to nursing practice [20, 23–26]. However, students’ attitudes toward AI are not shaped solely by individual awareness; they are also influenced by the cultural contexts in which students are embedded. Accordingly, cultural considerations should be taken into account when examining students’ attitudes toward AI [27].

In the process of adopting AI technologies, cross‐national differences in cultural norms, the structural characteristics of healthcare systems, and the educational levels of nurses emerge as key determining factors [28]. In Türkiye, where this study was conducted, digital transformation and technological integration within the health sector have accelerated in recent years [29], providing nursing students with increasing opportunities to engage with AI and other health technologies. Nevertheless, the extent to which these opportunities are utilized and technologies are adopted appears to be closely linked to Türkiye’s cultural and societal dynamics, as prevailing societal attitudes toward technology may shape nursing students’ perceptions of and attitudes toward AI. Therefore, cultural context should not be overlooked when evaluating undergraduate nursing students’ attitudes toward AI [30, 31].

In Türkiye, nursing education is delivered as an intensive four‐year undergraduate program in accordance with national regulations and may be accredited by the Association for Evaluation and Accreditation of Nursing Education Programs (HEPDAK). This educational structure aims to promote the early development of clinical competence, professional responsibility, and a patient‐centered approach to care among nursing students [32, 33]. When the structural characteristics of the Turkish education system and cultural context are taken into consideration, nursing students’ attitudes toward AI appear to be shaped predominantly by perceptions of usefulness, ethical appropriateness, and support for care quality. Consistent with this perspective, Sergeeva et al. [30] reported that students in educational environments with similar contextual characteristics perceive AI as a supportive tool aligned with professional values.

This observation is further supported by empirical evidence from the Turkish context. In a study conducted by Saydamlı et al. [34], 68% of students stated that the use of AI would positively influence their professional outlook by increasing the time allocated to patient care and treatment, clearly highlighting the perception of AI as a complementary component of nursing practice. Moreover, the belief that technology can assume responsibility for routine tasks such as data entry, documentation, and logistical processes—thereby allowing nurses to devote more time to direct patient interaction, compassionate care, and the strengthening of human connection—provides an important cultural and professional framework for understanding students’ positive and ethically grounded attitudes toward AI, as well as their intention to use AI‐based technologies [35, 36].

While robust technological infrastructures in developed countries facilitate the rapid integration of AI applications into healthcare systems, resource constraints and limited levels of digital literacy in developing countries may hinder this process [37]. Accordingly, nursing, health, and social policies that support the use of AI should be designed through inclusive strategies that explicitly account for these structural and cultural differences [38]. Therefore, nursing educators should adopt innovative pedagogical approaches that integrate AI into the curriculum according to students’ cultural characteristics to strengthen their competencies and encourage constructive attitudes towards the use of AI [39].

Nurses play a critical role in providing high‐quality care, and effective time management is a key factor influencing care quality. Nurses who lack time management skills may hinder team efficiency and fail to meet the demands of complex healthcare systems [40]. Nursing students likewise need to develop the ability to analyze how they use their time and identify areas for improvement [41]. Başak et al. [42] found that nursing students require enhanced time‐planning skills, that older students perform better in this regard, and that higher time management scores are associated with greater academic achievement. Effective time management is essential for nursing students’ academic success and their transition into professional roles [43]. This issue becomes even more critical for students who are exposed to an intensive curriculum, such as those enrolled in nursing education program. The use of AI tools may be considered a promising approach to supporting the development of time management skills among nursing students. Students can employ AI‐based tools in ways that align with their individual characteristics to facilitate rapid access to information, bridge the gap between theory and practice, manage time constraints, and support participation in classroom teaching, as well as in their daily activities [44, 45].

Integrating time management education into the nursing curriculum and emphasizing its importance in clinical practice may enhance students’ preparedness for professional responsibilities and the healthcare system. The ability to manage nursing workloads safely and in a timely manner is a learned process that requires structured support, highlighting the need for targeted interventions aimed at strengthening time management competencies [46]. Given the efficiency and speed‐enhancing potential of AI, a positive attitude toward this technology may also contribute to improved time management among nursing students. However, in today’s AI‐driven healthcare environment, it is increasingly important to understand not only nursing students’ attitudes toward AI but also how they engage with AI‐based tools in their academic and clinical learning processes and how the use of these technologies may influence their ability to manage time effectively. An examination of the available literature reveals a clear gap, as no study has directly investigated the association between these two important variables, emphasizing the necessity for further in‐depth research. Accordingly, this study aimed to describe undergraduate nursing students’ attitudes toward AI and their time management skills and to examine the relationship between these variables.

### 1.1. Research Questions

1. What are the levels of nursing students’ attitudes toward AI?
2. What are the levels of nursing students’ time management skills?
3. Is there a relationship between nursing students’ attitudes toward AI and their time management skills?

## 2. Methods

### 2.1. Design

A cross‐sectional study design was employed in accordance with the descriptive and relational objectives of the study. Data collected between September and December 2024 were used to examine undergraduate nursing students’ attitudes toward AI and their time management skills, as well as the relationship between these variables at a single point in time without implying causality. The study followed the Strengthening the Reporting of Observational Studies in Epidemiology (STROBE) guidelines (Appendix 1).

### 2.2. Sample

The population of this study consists of a total of 698 active undergraduate students in the first, second, third, and fourth years studying in the Nursing Department of a state university located in the northwest of Turkey. The sample size of the study was calculated using the G∗power 3.1 program; with a power value of 0.30 and an error level of 0.05, it was determined that a minimum of 340 students should be included in the sample [47]. To compensate for potential data loss due to incomplete questionnaires and exclusions during data screening, an oversampling strategy of approximately 5%–15% was applied, as recommended in survey‐based and cross‐sectional research [48, 49].

The inclusion criteria for the study were (1) being actively enrolled as an undergraduate student in the Nursing Department (first, second, third, or fourth‐year undergraduate nursing student) at the study institution during the data collection period, (2) voluntarily agreeing to participate in the study and providing written informed consent, and (3) being able to understand and complete the survey instruments in Turkish. Both domestic and international undergraduate nursing students who met the above criteria were included in the study.

The exclusion criteria for the study were (1) submitted incomplete or missing survey data and (2) declined to participate or withdrew from the study at any stage of data collection. No clinical assessment or self‐report screening related to psychiatric diagnoses was conducted as part of the study; therefore, students were not excluded based on psychiatric conditions.

### 2.3. Data Collection Tools

Following approval from the ethics committee and faculty administration using a face‐to‐face survey method, the surveys were administered to undergraduate nursing students who volunteered to participate in designated nonclassroom settings within the faculty building. To avoid disruption of academic activities, data collection was conducted during students’ free time outside scheduled class hours. Communication with students was established through face‐to‐face invitations and brief verbal explanations prior to participation. Throughout the data collection period, there was no teaching or assessment relationship between the researchers and the participating students. Each survey session was completed individually under the supervision of a researcher and took approximately 10–15 min.

#### 2.3.1. Individual Identification Form

This form, created by researchers, contains participants’ demographic information such as age, gender, class, academic grade point average, and their opinions on AI.

#### 2.3.2. General Attitudes Towards AI Scale (GAAIS)

Developed by Schepman and Rodway [50], this scale has a five‐point Likert structure (1 = strongly disagree‐5 = strongly agree). The scale was adapted into Turkish by Kaya et al. [51]. In the Turkish version, items 1–12 form the positive subscale, while items 13–20 form the negative subscale. Negative items are reverse scored. A higher score on the scale indicates a more positive attitude toward AI. In the original study, the Cronbach’s alpha value for the positive subscale was determined to be 0.88, and for the negative subscale, it was 0.83, indicating good internal consistency reliability [50]. In the Turkish adaptation study, the Cronbach’s alpha value for the positive subscale was determined to be 0.82, and the Cronbach’s alpha value for the negative subscale was determined to be 0.84 [51]. In this study, Cronbach’s alpha value of the GAAIS was determined to be 0.834, Cronbach’s alpha value of the positive subscale was determined to be 0.873, and Cronbach’s alpha value of the negative subscale was determined to be 0.860.

#### 2.3.3. Time Management Questionnaire (TMQ)

The validity and reliability of the TMQ, originally developed by Britton and Tesser [52], were established for the Turkish context by Alay and Koçak [53]. The TMQ comprises 27 items grouped into three subscales: Time Planning (TP) with 16 items, Time Attitudes (TA) with 7 items, and Time Wasters (TW) with 4 items. Items are rated on a five‐point Likert scale ranging from 1 = never to 5 = always. Items 16, 18, 22, 23, 24, 25, 26, and 27 are reverse‐scored, with responses coded from 1 = always to 5 = never. The total score ranges from 27 to 135, with higher scores indicating better time management. In the Turkish adaptation, the overall Cronbach’s alpha coefficient of the TMQ was reported as 0.87 [53]. In the present study, the internal consistency coefficients were 0.843 for the total scale, 0.883 for the TP subscale, 0.484 for the TA subscale, and 0.534 for the TW subscale.

### 2.4. Data Analysis

The data obtained as a result of the research were analyzed using the Statistical Package for the Social Sciences (SPSS) (IBM Corp., Armonk, NY, USA) version 25 program. The normality of continuous variables was tested with the Shapiro–Wilk test [54]. Descriptive statistical methods (number, percentage, min–max values, mean, and standard deviation) were used to evaluate the data. For data with a normal distribution, an independent *t*‐test was used to compare quantitative data between two independent groups, and a one‐way analysis of variance was used when comparing more than two independent groups. When a difference was found, Bonferroni was used to find the group that caused the difference. Pearson correlation analysis was used to test the relationship between variables.

### 2.5. Ethical Considerations

Permission was obtained from the Sakarya University Social and Human Sciences Ethics Committee to conduct the research (E‐61923333‐050.99‐354216, 04/18/2024). After the ethics committee approval, permission was also obtained from the faculty to administer the survey (E‐81791324‐051‐356158). Students were informed about the study and included in the study on a voluntary basis. The entire implementation process of the study was carried out in accordance with the Helsinki Declaration and based on an ethical framework.

## 3. Results

### 3.1. Comparison of Scales Mean Scores According to Demographic Variables

The distribution of participants’ demographic characteristics, the GAAIS, and the comparison of dimension scores and TMQ scores are presented in Table 1. It was seen that 58.9% of the participants are 21 years old and above, 83.3% are female, 32.5% are in the first grade, and 51.9% have a GPA of 2.50 and above.

**TABLE 1:** Comparison of participants’ mean scores of GAAIS and TMQ according to demographic variables (*n* = 360).

Variables		*n*	%	Positive subscale	Negative subscale	GAAIS	TP	TA	TW	TMQ
				X̅ ± SS (min–max)	X̅ ± SS (min–max)	X̅ ± SS (min–max)	X̅ ± SS (min–max)	X̅ ± SS (min–max)	X̅ ± SS (min–max)	X̅ ± SS (min–max)
Age	Under 21	148	41.1	43.97 ± 6.55	22.96 ± 5.88	69.01 ± 8.46	49.43 ± 9.96	21.80 ± 3.66	12.93 ± 2.76	84.17 ± 12.58
	21 years old and above	212	58.9	43.85 ± 7.11	23.00 ± 5.66	68.85 ± 9.72	50.21 ± 10.48	22.56 ± 3.16	12.83 ± 2.96	85.61 ± 12.63
	Test value			0.159^∗∗^	−0.058^∗∗^	0.155^∗∗^	−0.707^∗∗^	−2.106^∗∗^	0.331^∗∗^	−1.064^∗∗^
	p			0.874	0.954	0.877	0.480	0.036	0.741	0.288
	D			0.017	−0.006	0.016	−0.075	−0.225	0.035	−0.114

Gender	Male	60	16.7	43.92 ± 7.79	22.25 ± 5.64	69.67 ± 10.26	47.38 ± 9.29	22.70 ± 3.24	12.28 ± 2.9	82.36 ± 11.10
	Female	300	83.3	43.89 ± 6.69	23.13 ± 5.77	68.77 ± 9.00	50.40 ± 10.39	22.16 ± 3.42	12.99 ± 2.85	85.55 ± 12.84
	Test value			0.024^∗∗^	−1.079^∗∗^	0.690^∗∗^	−2.087^∗∗^	1.118^∗∗^	−1.740^∗∗^	−1.792^∗∗^
	P			0.981	0.281	0.490	0.038	0.264	0.083	0.074
	D			0.003	−0.152	0.097	−0.295	0.158	−0.246	−0.253

Grade	1st grade	117	32.5	44.50 ± 6.26	22.65 ± 5.67	69.85 ± 8.40	49.47 ± 9.89	21.90 ± 3.79	13.43 ± 2.69	84.82 ± 12.66
	2nd grade	79	21.9	42.20 ± 7.40	24.14 ± 5.73	66.06 ± 9.50	49.27 ± 11.52	22.01 ± 2.99	11.94 ± 2.91	83.24 ± 13.29
	3rd grade	99	27.5	43.65 ± 6.92	23.09 ± 5.40	68.56 ± 9.06	51.13 ± 10.01	22.76 ± 3.08	12.71 ± 2.75	86.61 ± 11.95
	Fourth grade	65	18.1	45.26 ± 6.91	22.00 ± 6.28	71.26 ± 9.75	49.52 ± 9.75	22.38 ± 3.50	13.21 ± 3.08	85.12 ± 12.61
	Test value			2.836^∗∗∗^	1.855^∗∗∗^	4.505^∗∗∗^	0.664	1.335	4.744	1.065
	P			0.038^∗^	0.137	0.004^∗^	0.575	0.263	0.003	0.364
	Bonferroni			4 > 2		1 > 2, 4 > 2			1 > 2, 4 > 2	
	*η* ^2^			0.023	0.015	0.037	0.006	0.011	0.038	0.009

Grade point average (GPA)	Under 2.50	173	48.1	43.58 ± 7.22	23.38 ± 6.00	68.21 ± 9.25	48.14 ± 10.52	21.40 ± 3.41	12.77 ± 2.81	82.32 ± 12.89
	2.50 and above	187	51.9	44.19 ± 6.55	22.62 ± 5.49	69.57 ± 9.15	50.46 ± 10.13	22.52 ± 3.34	12.90 ± 2.90	85.89 ± 12.41
	Test value			−0.832^∗∗∗^	1.256^∗∗∗^	−1.405^∗∗∗^	−1.845	−2.706	−0.372	−2.318
	P			0.406	0.210	0.161	0.066	0.007	0.710	0.021
	D			0.151	0.106	0.046	−0.226	−0.331	−0.045	−0.284

Total		360	100	43.90 ± 6.87 (22–60)	22.98 ± 5.75 (8–40)	68.92 ± 9.21 (43–96)	49.89 ± 10.26 (19–78)	22.25 ± 3.39 (10–34)	12.87 ± 2.88 (4–20)	85.02 ± 12.61 (45–115)

*Note:* ^∗^ *p* < 0.05, ^∗∗^An independent *t*‐test and ^∗∗∗^a one‐way analysis of variance was performed. d: Cohen’s d, *η* ^2^: Eta squared.

Abbreviations: GAAIS, General Attitudes Toward Artificial Intelligence Scale; TA, Time Attitudes; TMQ, Time Management Questionnaire; TP, Time Planning; TW, Time Wasters.

The mean positive subscale was 43.90 ± 6.87, the mean negative subscale was 22.98 ± 5.75, the mean score of GAAIS was 68.92 ± 9.21, the TP mean score was 49.89 ± 10.26, the TA mean score was 22.25 ± 3.39, the TM mean score was 12.87 ± 2.88, and the TMQ mean score was 85.02 ± 12.61 (Table 1).

Statistically significant differences across grade levels were observed for the positive subscale (*p* = 0.038, *η* ^2^ = 0.023) and for GAAIS scores (*p* = 0.004, *η* ^2^ = 0.037), both indicating small effect sizes. Bonferroni was applied to find the group that created the difference. It was seen that the positive subscale and GAAIS scores of fourth‐year nursing students were higher than those of second‐year nursing students. It was seen that the GAAIS scores of first‐year nursing students are higher than those of second‐year nursing students (Table 1).

There was no statistically significant difference in the positive subscale, negative subscale, and GAAIS score according to the participants’ age, gender, and GPA (*p* > 0.05).

There was a statistically significant difference in TA scores according to participants’ age (*p* = 0.036), with a small effect size (*d* = −0.225). Participants aged 21 and above have higher TA scores than participants under the age of 21. Statistically significant difference in TP scores according to participants’ gender (*p* = 0.038), with a small effect size (*d* = −0.295). Female participants’ TP scores are higher than those of male participants.

Statistically significant difference in TW scores of participants according to grade (*p* = 0.003), with a small effect size (*η* ^2^ = 0.038). Bonferroni was applied to find the group that created the difference. First‐year and fourth‐year nursing students’ TW scores are higher than those of second‐year nursing students.

Statistically significant differences across participants’ overall GPA were observed for TA (*p* = 0.007, *d* = −0.331) and for TMQ scores (*p* = 0.021, *d* = −0.284), both indicating small effect sizes. Participants with a GPA of 2.50 and above have higher TA scores than participants with a GPA below 2.50 (Table 1).

There was no statistically significant difference between participants’ TP, TW, and TMQ scores based on their age; between their TA, TW, and TMQ scores based on their gender; or between their TP, TA, and TMQ scores based on their grades. There was no statistically significant difference between TP, TW, and TMQ scores based on GANO (*p* > 0.05).

### 3.2. Analysis of the Relationship Between GAAIS and TMQ

Pearson correlation was applied to test the relationship between the scales used in the study. As a result, a statistically significant and positively directed relationship was found between the TP and the positive subscale (*r* = 0.137, *p* = 0.009) and GAAIS (*r* = 0.128, *p* = 0.015). A statistically significant and negatively directed relationship was observed between the TW and the negative subscale (*r* = −0.119, *p* = 0.024). A statistically significant and positively correlated relationship was observed between the TMQ and GAAIS (*r* = 0.119, *p* = 0.024) (Table 2).

**TABLE 2:** Correlation analysis between GAAIS, TMQ, and subscales.

Variables	Positive subscale		Negative subscale		GAAIS	
	r	p	r	p	r	p
TP	0.137	0.009^∗^	−0.042	0.430	0.128	0.015^∗^
TA	−0.049	0.354	−0.068	0.201	0.006	0.916
TW	−0.022	0.681	−0.119	0.024^∗^	0.058	0.274
TMQ	0.093	0.077	−0.079	0.134	0.119	0.024^∗^

*Note:* The Pearson correlation test was applied.

Abbreviations: GAAIS, General Attitudes Toward Artificial Intelligence Scale; TA, Time Attitudes; TMQ, Time Management Questionnaire; TP, Time Planning; TW, Time Wasters.

^∗^ *p* < 0.05.

## 4. Discussion

This study aimed to describe undergraduate nursing students’ attitudes toward AI and their time management skills and to examine the relationship between these variables. The findings were discussed under three overarching themes in alignment with the relevant literature.

### 4.1. Attitudes Towards AI

Nursing students’ attitudes toward AI provide important insights into how they are likely to adopt, utilize, and integrate these technologies into patient care [55]. The findings of the present study demonstrated that nursing students exhibited a high level of positive attitudes toward AI, which is consistent with results reported in studies conducted within similar cultural contexts [16, 56]. Comparable findings have also been reported in studies from other countries, indicating that the present results are aligned with the broader international literature on attitudes toward AI in nursing and healthcare education [20, 57, 58]. Consistent with the literature, students in nursing and other health‐related disciplines generally display favorable attitudes toward AI, often influenced by factors such as grade level, clinical experience, and AI literacy [59, 60]. In the present study, fourth‐year nursing students scored higher on both the positive attitude dimension and overall attitude scale compared to second‐year students. Similarly, first‐year students’ overall attitude scores were higher than those of second‐year students (Table 1).

Previous research supports these findings, indicating that upperclassmen exhibit more positive attitudes and stronger intentions to integrate AI into practice [59, 60]. A study conducted in Korea also reported that upper‐level students had more favorable attitudes toward AI than lower‐level peers [61]. In contrast, Lukić et al. [58] found lower overall attitude scores among first‐year nursing students. This discrepancy may be explained by fourth‐year students’ greater exposure to AI‐related content, their higher technological competence, and their use of AI tools in educational and clinical contexts. Additionally, receiving formal AI education or direct exposure to AI applications has been associated with more positive attitudes [21, 62, 63]. Conversely, other studies have reported more positive attitudes among second‐year students [64], suggesting that both increased experience among upperclassmen and greater technological familiarity among lower‐level students can shape attitudes positively [58].

In the present study, no significant difference was observed in attitudes toward AI according to gender. However, previous studies have reported gender as a significant factor influencing attitudes toward AI [34, 65, 66]. Specifically, Saydamlı et al. [34] found that female nursing students had significantly higher positive attitude scores toward AI compared with their male counterparts. In contrast, Çelik and Dönmez [65] reported that male university students demonstrated significantly higher AI attitude scores than females. Similarly, Çatman et al. [66], in a large‐scale study involving 1402 students, confirmed that male students exhibited higher levels of interest in AI. These divergent findings may reflect differences between the care‐oriented and pragmatic perspectives commonly observed among female students in health‐related fields and the technology‐oriented interest often reported among male students in the general student population.

The adaptation of the new generation to AI‐supported technologies and their ability to use these technologies effectively are of considerable importance for the future. Accordingly, AI has been increasingly incorporated into curricula as foundational and theoretical course content across different educational levels to support digital competence and technological readiness [6, 67].

From a practical standpoint, nursing educators should integrate AI‐related content into undergraduate and graduate curricula to foster students’ knowledge, confidence, and engagement with emerging technologies [5]. Such integration may enhance students’ ability to manage, utilize, and contribute to AI initiatives in healthcare. As a result, it can be anticipated that nursing students will increasingly use and follow AI‐based technologies both in their studies and in their future clinical practice.

AI technology offers numerous advantages across multiple domains, including data processing, storage, reporting, interpretation, standardization, time efficiency, cost reduction, and the prevention of human‐related errors in the delivery and management of healthcare services [6, 68–70]. In this context, organizations increasingly require a workforce that is capable of understanding how to use technology effectively and how to capitalize on the opportunities offered by AI [71]. Therefore, it is essential that nursing students, as future healthcare professionals, acquire the competencies necessary to use AI‐based tools effectively and responsibly in professional practice.

### 4.2. Time Management Skills

This study found that nursing students possessed high levels of time management skills. Similar results have been reported in previous research, which generally found nursing students’ time management abilities to range from moderate to high [43, 72–75]. The finding that older students had higher TA scores aligns with existing evidence showing that time management tends to improve with age and experience [43, 76]. This trend may be explained by older students’ greater exposure to academic responsibilities and increased familiarity with workload planning. As students’ progress through their educational programs, practical workshops and programs should be planned to help them adopt effective strategies for prioritizing tasks, managing deadlines, and balancing academic and personal demands [77]. From an educational perspective, this finding underscores the importance of providing targeted time management support to younger students, particularly during the early years of nursing education, to promote the development of these skills at an earlier stage [78].

Gender‐based differences were also observed: female students had significantly higher TP scores than male students (*p* < 0.05). This finding mirrors previous research in Turkey, which demonstrated that female students outperform males in TP and overall time management [43]. This difference may reflect women’s greater tendency toward organization and structured planning in socialization processes and role expectations. In the context of nursing education, where time‐sensitive tasks, multiple assignments, and clinical obligations are common, such tendencies may facilitate more effective prioritization and scheduling. However, as time management skills are shaped not only by gender but also by individual experiences, educational leadership, academic demands and contextual factors [43, 77–79], this interpretation should be approached with caution.

Nursing education involves intensive theoretical and clinical components that require effective time use. Time management is therefore critical to balancing academic and social demands and is closely linked to both academic performance and future professional competence [74]. Gündoğdu et al. [79] also found that demographic factors such as grade level, gender, and income significantly influence time management skills.

In this study, first‐ and fourth‐year students demonstrated better time management skills than second‐year students. This aligns with research showing that students in higher grades manage their time more effectively [80]. The superior time management of upperclassmen may be attributed to their internship experiences and their proximity to professional transition, which demand greater scheduling discipline and prioritization.

### 4.3. Relationship Between Attitudes Toward AI and Time Management Skills

AI enhances efficiency, reduces administrative burdens, and enables the delivery of personalized learning experiences [81]. AI technologies have been shown to enhance time management by automating routine tasks, increasing efficiency, and improving productivity [82]. They also facilitate clinical documentation by structuring data and offering real‐time decision support [83, 84]. Integrating AI into nursing education and clinical training may therefore promote students’ ability to manage their time more effectively [59, 85, 86].

This study demonstrated a statistically significant but weak positive correlation between nursing students’ overall attitudes toward AI and their time management skills (*r* = 0.119, *p* = 0.024) (Table 2). This finding suggests that more positive attitudes toward AI are associated with time management skills to a limited extent; however, the strength of this relationship is weak and does not indicate a strong or determinative effect. Therefore, it would not be appropriate to infer that attitudes toward AI directly or substantially enhance students’ time management abilities. Nevertheless, in line with the existing literature, the perception of AI technologies as tools that support workflow and enhance efficiency in educational and clinical contexts may point to an indirect and context‐sensitive relationship between students’ attitudes toward these technologies and the ways in which they regulate their learning processes [87]. The weak correlation coefficient further indicates that this relationship is likely shaped by multiple factors, including individual learning habits, levels of digital literacy, the structure of educational programmes, and the frequency of exposure to AI‐based applications.

In this context, the potential effects of AI on nursing students’ time management skills cannot be explained solely by attitudes toward AI; rather, they should be considered in conjunction with factors such as pedagogical design, instructor guidance, and the manner in which technology is integrated into the learning environment. This finding underscores the need for caution against assuming that positive attitudes toward AI will automatically translate into improvements in academic or clinical performance, and it highlights the importance of future longitudinal and experimental studies to clarify these relationships.

Planned educational interventions and pedagogical strategies aimed at supporting students’ adaptation to AI technologies may contribute indirectly to the development of time management skills, in addition to enhancing technological competencies [59]. Accordingly, nursing curricula should be structured to encompass not only technical knowledge of AI but also AI literacy, ethical awareness, critical appraisal skills, and guiding principles for the safe use of AI in clinical practice.

Introducing AI education at early stages of undergraduate programmes may enable students to perceive these technologies as a natural component of their learning processes. Such an approach may facilitate more systematic planning of learning activities and more conscious management of time as students encounter increasing theoretical and clinical responsibilities. Nevertheless, it should be acknowledged that the effects of these educational approaches on time management are not direct but are shaped by contextual factors, including the learning environment, individual learning styles, and institutional support. Therefore, ensuring pedagogical coherence and adherence to principles of progressive learning is critical in the design of AI‐based educational content.

Joint assessment of nursing students’ attitudes toward AI and their time management skills provides complementary insight into how educational processes may be structured to better support students’ transition to professional practice. Consistent with the preceding discussion, the findings indicate that positive attitudes toward AI are associated with, rather than determinative of, students’ approaches to organizing learning demands and managing time. This suggests that openness to AI may operate as an indirect and facilitating factor, shaped by pedagogical design, learning context, and individual self‐regulatory capacities, rather than as an independent driver of time efficiency or professional readiness.

In this context, enhancing the scope and impact of AI applications in nursing requires their integration into education and health policy through a holistic framework that balances technological innovation with ethical, pedagogical, and professional considerations. Such an approach supports the view that AI should be embedded as a supportive educational resource within nursing curricula, contributing to the development of adaptable, reflective, and time‐conscious future nurses, without overstating its role as a singular solution to complex educational and clinical challenges.

### 4.4. Limitations of the Study

This study has several limitations that should be considered when interpreting the findings. First, its cross‐sectional design precludes causal inferences between nursing students’ attitudes toward AI and their time management skills. Longitudinal or experimental studies are recommended to explore how these variables interact over time and whether educational interventions can enhance both attitudes and time management abilities. Second, the data were collected from a single institution using self‐reported questionnaires, which may introduce response bias and limit generalizability. Thirdly, the Cronbach’s alpha coefficients for the TA subscale and the TW subscale are below the generally accepted threshold value. Although these values are comparable to those reported in some previous studies, findings related to these subscales should be interpreted with caution. Future research may benefit from further psychometric evaluation of these subscales or the use of alternative instruments to more robustly assess concepts related to time management. Considering the faculty infrastructure and facilities, economic status, and access to technological devices for AI among nursing students, the findings of the study are limited to the country in which it was conducted and should be interpreted within the framework of Turkish culture.

### 4.5. Recommendations for Future Research and Implications

Future studies should include larger and more diverse samples from different regions and educational settings to strengthen external validity. The study focused on general attitudes toward AI rather than specific dimensions such as AI literacy, ethical awareness, or practical competence. Subsequent research could examine these subcomponents in greater detail to identify the mechanisms through which AI readiness influences learning behaviors and professional development. Finally, the study did not include objective indicators of time management (e.g., academic performance and clinical task completion), which could complement self‐reported measures. Incorporating mixed‐method designs or objective digital metrics in future studies would provide a more comprehensive understanding of how AI‐related attitudes translate into effective time use and professional preparedness.

## 5. Conclusions

This study concluded that nursing students demonstrated a high level of positive attitudes towards AI and high levels of time management skills scores. This study provided important insights into the relationship between undergraduate nursing students’ attitudes towards the use of AI and their time management skills. A positive relationship was identified between students’ general attitudes towards AI and their ability to manage time effectively. This relationship supports existing evidence suggesting that AI can enhance time efficiency in clinical and educational settings. To strengthen these findings, controlled intervention and longitudinal studies are needed to determine the mechanisms and long‐term effects of AI‐based improvements in time management. Incorporating modules on AI literacy, ethical use, and patient safety into undergraduate nursing curricula is recommended to ensure that students can integrate AI tools responsibly and effectively.

## Author Contributions

Ramazan Bozkurt: methodology, conceptualization, validation, formal analysis, data curation, investigation, resources, visualization, writing–original draft, writing–review and editing, and software.

Musa Can Özdoğan: conceptualization, validation, formal analysis, data curation, investigation, resources, writing–original draft, writing–review and editing, project administration, and visualization.

## Funding

This work was supported by the Scientific and Technological Research Council of Türkiye (TÜBİTAK) 2209‐A University Student Research Projects Support Program (1919B012314152, 2023/2).

## Ethics Statement

Approval for the conduct of the research was received from the Sakarya University Social and Human Sciences Ethics Committee (E‐61923333‐050.99‐354216, 18.04.2024). Permission was also obtained from the faculty to conduct the research (E‐81791324‐051‐356158). Students were informed about the research before data collection, they were included in the study on a voluntary basis, and the research was conducted in accordance with the Helsinki Declaration.

## Conflicts of Interest

The authors declare no conflicts of interest.

## Supporting Information

STROBE Statement—Checklist of items that should be included in reports of cross‐sectional studies.

## Supporting information


**Supporting Information** Additional supporting information can be found online in the Supporting Information section.

## Data Availability

The dataset can be shared upon request for use in further research.
